# Heavy metals in essential oils proposed for the modification of footwear lining leather—Quality management and product safety

**DOI:** 10.1371/journal.pone.0325766

**Published:** 2025-06-26

**Authors:** Elżbieta Bielak

**Affiliations:** Department of Non-food Product Quality and Safety, Institute of Quality Sciences and Product Management, Krakow University of Economics, Krakow, Poland; University of the Witwatersrand Johannesburg, SOUTH AFRICA

## Abstract

Due to their antiseptic action, essential oils can be incorporated into leather in order to improve the hygienic properties of footwear by reducing microbial growth and multiplication in the interior. This paper presents the results of analyses of the contents of heavy metals, i.e., As, Cd, Cu, Ni and Pb in cinnamon, eucalyptus, oregano, manuka and thyme oils proposed for modification of lining leather. Experiments carried out using atomic absorption spectrometry were designed to evaluate the impact of the addition of these oils on the quality and safety of footwear considering the possible presence of toxic metals. In the context of the limits for metal contents in products set out in the European REACH Regulation, Oeko-Tex® Leather Standard by Oeko-Tex® Association, *Restricted Substances List* by AFIRM Group and *List of Restricted Substances in Shoes* by CADS Association, it was found that the oils tested were characterised by low levels of As (thyme, oregano and eucalyptus oils: not detected, other oils: 0.002 mg/kg – 0.015 mg/kg), Cd (0.001 mg/kg – 0.022 mg/kg), Cu (0.06 mg/kg – 0.184 mg/kg), Ni (0.025 mg/kg – 0.144 mg/kg) and Pb (0.06 mg/kg – 0.097 mg/kg). Therefore, their application to lining leather should not impair the quality of footwear understood in terms of its safety for the user. Given the limited number of analyses of heavy metal contents in essential oils of various applications, in the context of the study conducted, which, although in small numbers, nevertheless confirmed the presence of metals in oils, it was concluded that such analyses should be conducted on a larger scale. In order to ensure the quality and safety of products enriched with essential oils, it is necessary to continuously monitor an important property of essential oils, namely their content of harmful heavy metals.

## Introduction

The group of heavy metals includes metals and metalloids that are characterised by relatively high densities; moreover, they can exhibit toxic effects even when present in low concentrations [1] and cause multiple organ damage [2]. The literature points to two sources of heavy metals in the environment. On the one hand, there is the natural origin, i.e., these metals can be found in soil, rocks or water; on the other hand, they are emitted as a result of human activities [3–5]. According to Djarmouni et al. [6], pollutants of anthropogenic origin are associated with, e.g., the use of herbicides and pesticides, waste disposal, and the activity of the metallurgical industry. However, the toxicity of heavy metals to the body will depend on the route, time and frequency of exposure. They can adversely affect the nervous, reproductive and immune systems [7].

Arsenic is an element that occurs naturally in the groundwater of selected countries in fairly large quantities. According to the World Health Organization [8] high toxicity is attributed to arsenic in its inorganic form. Exposure to this element is mainly related to the consumption of water and food contaminated with it. In terms of industrial application, arsenic is used in the production of, e.g., pigments, paper, textiles and leather (during tanning); it also acts as an alloying agent. Long-term exposure to this element, mainly through consumption of contaminated water and food, can cause skin lesions and cancer, as well as cardiovascular disease or diabetes. Heavy metals also include cadmium, which occurs naturally in water, soil and minerals [9]. Its presence in the environment as a result of human activity is linked to the mining and metallurgical industries, the production of nickel-cadmium batteries, dyes, the stabilisation of plastics [10], and it can also be found in biocides and fertilisers [11]. Cadmium is considered to be one of the most toxic elements to which humans can be exposed in both occupational and natural environments. Absorbed into the body through food and inhalation, it accumulates in the body for long years [9]. With chronic exposure, it can contribute to a variety of cancer types affecting organs such as the lungs, pancreas, kidneys, breasts and prostate. The liver and kidneys are particularly vulnerable to its toxic effects. Cadmium also causes DNA damage [12]. Copper occurs naturally in the environment in low concentrations in the Earth’s crust and seawater. It does not decompose, but when released into the air, soil or water, it bonds with other molecules thereby its toxicity is reduced. The metal is widely used in industry, e.g., in the manufacture of cement, concrete products, paper, glass, ceramics and textiles [13]. Too much copper introduced into the human body causes oxidative stress, reduced cell proliferation and DNA damage [14]. Nickel is an element occurring naturally in soil and groundwater. Industrial applications involve the use of this metal in electroplating and the production of alloys, stainless steel and rechargeable batteries. Nickel carbonyl is considered to be the most hazardous form of nickel for humans. Occupational exposure to this compound results in respiratory irritation. Possible outcomes of chronic exposure may include sinusitis or skin inflammation, asthma and even nasal and lung cancer [15]. It is estimated that approx. 5–13% of the world’s population is allergic to nickel [16]. There is another toxic heavy metal present in the Earth’s crust, i.e., lead. In industry it is mainly used in the manufacture of batteries used in motor vehicles. Furthermore, this element can be found in a wide variety of products, including lead crystal glassware, ceramic glazes, paints, toys and jewellery. The presence of lead in water is due to the fact that it is supplied through pipes made of this metal or pipes that are joined with lead-based solder. As a result of exposure, the element enters the brain, liver, kidneys and bones. In adults, it contributes to cardiovascular disorders, increases blood pressure and damages the kidneys. This metal is very dangerous to the developing foetus and can affect the development of the brain and nervous system [17].

Due to the multitude of applications, heavy metals are present in many non-food products with which consumers come into direct contact every day. An example of products frequently tested for heavy metals, because of their toxicity, are cosmetics [18–22] in the case of which consumer safety is particularly important due to the direct application to the skin. Other non-food products for which it is essential that they do not pose a chemical risk to users, in view of the way in which they are used and how frequently, are items of clothing and footwear, worn by millions of people worldwide every day. The subject of exposure to heavy metals due to their presence in both adult and children’s clothing is an ever-present issue, repeatedly addressed in various publications that have appeared in recent years. It has been discussed by, among others, Bielak and Marcinkowska [23], Chen et al. [24], Herrero et al. [25], Nyamukamba et al. [26], Panhwar et al. [27], Sima [28]. Bielak and Marcinkowska [23] conducted some analyses that concerned not only the assessment of heavy metal contents in clothing, but also in materials intended for internal and external components of footwear, i.e., leathers and artificial leathers. The analyses proved that commercially available raw materials of this type are of questionable quality and may pose a risk to consumer health and safety due to excessive chromium content. The presence of this metal, as well as copper, cadmium, nickel, lead, and other harmful compounds, such as carcinogenic aromatic amines from azo dyes, ortho-phenylphenol, 2,4,6-tribromophenol, dimethylfumarate and formaldehyde in footwear made in different countries around the world has been demonstrated by Prevodnik [29]. His research confirmed the fact that hazardous chemicals can be present in footwear regardless of price, manufacturer or where it was produced. It is therefore necessary to carry out research to verify the quality of raw materials and components used in footwear production to ensure the quality and safety of the final product. Quality control of materials such as leather and textiles, or finished footwear components, e.g., soles, midsoles or laces, provides for a number of tests on their various properties, among which is the assessment of the contents of harmful substances, including heavy metals [16].

The problem of the harmful effects of heavy metals in connection with technological processes for the production of raw materials and products, and their use does not only concern humans, but also the natural environment. In the context of the tanning industry, which supplies raw material to the footwear industry, the focus should be primarily on the aforementioned chromium. Chromium (III) compounds are used in leather tanning. This element will therefore be present not only in leather and leather products, but also in the waste generated by tanneries. Inadequate management/disposal of such waste leads to environmental pollution by toxic chromium as pointed out by Senthil [30]. Accordingly, his study proposed an effective use of solid wastes by converting them into useful resources, i.e., making a regenerated flexible sheet – a composite based on chrome waste powder, cardboard waste fiber and protein binder. The developed material is an alternative to natural leather and, according to the author, can be used in the production of, e.g., footwear. In another study by the same researcher [31], chrome-containing leather waste was used to prepare a polymerized electrolytic solution in order to verify the possibility of using it in sustainable energy production. Some other solutions that are also being proposed for the tanning industry aim to prevent heavy metals from entering the environment. In 2022 scientists from Turkey, India and the US used microfibrillated cellulose, tea leaf microparticles and poly(vinyl) alcohol to prepare a fibrous nanofilter capable of removing heavy metals from tannery wastewater. In their experiments they demonstrated its effectiveness with regard to Cr (VI) [32]. During leather production processes the Cr (III), which is used for tanning, can, under favorable conditions (e.g., UV irradiation or contact with alkaline solutions), oxidize to a much more dangerous form, namely the Cr (VI) mentioned above. Both Cr (III) and Cr (VI) are responsible for allergic contact dermatitis in people allergic to chromium who come into contact with products tanned using this metal [33]. Of course, other heavy metals that may be present in footwear materials are also a problem. The toxicity of footwear soles in terms of the impact of the chemicals they contain on the aquatic environment was assessed by Ingre-Khans et al. [34]. Their experiments proved that the toxicity of the footwear components tested was mainly due to the presence of zinc in the material analysed.

The aim of this study was to quantify the contents of five selected heavy metals, i.e., arsenic, cadmium, copper, nickel and lead, in the essential oils – cinnamon, eucalyptus, oregano, manuka and thyme, which have been proposed for the enriching of lining leather because of their potential to improve the hygienic properties of footwear. The results of atomic absorption spectrometry analyses were related to the current limits for the permissible contents of the indicated metals in products, including leather, contained in the European REACH Regulation [35], Oeko-Tex® Leather Standard by Oeko-Tex® Association [36], *Restricted Substances List* by AFIRM Group [37] and *List of Restricted Substances in Shoes* by CADS Association [38]. This allowed the evaluation of the possible impact of the addition of the above-mentioned oils on the quality and safety of leather, which is the raw material for the production of footwear inner components, and thus the final product. In the world literature, there are very few reports of studies on the contents of heavy metals in the types of essential oils selected for leather enriching despite their wide use in many other commercially available industrial products. In this context, it is therefore reasonable to draw attention to the problem and to the need to verify the properties of plant raw materials of this type, related to the possible contents of toxic heavy metals, which is particularly important when oils are used in products with which consumers have direct and/or frequent contact.

## Materials and methods

### Essential oils tested for the presence of heavy metals

**Fig 1:**
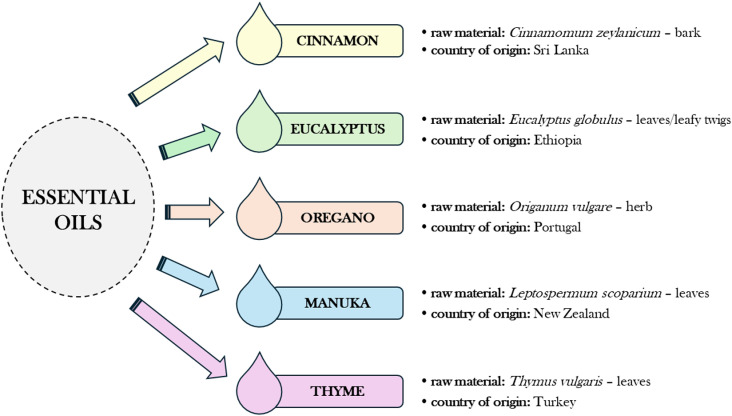
Characteristics of the research material – essential oils. Source: Author’s own research.

Five plant essential oils, i.e., cinnamon, eucalyptus, oregano, manuka and thyme oils were tested for the contents of selected heavy metals. Fig 1 shows their characteristics including the part of the plant from which the oil was extracted with the Latin name of the species as well as the country in which the oil was produced. The study material came from Asia, Africa, Europe and Oceania. According to the declarations of the manufacturers, each of the oils listed above was obtained by steam distillation. For the purpose of the study, the oils were donated by the Laboratory of Industrial and Experimental Biology at the State Higher Vocational School Stanisław Pigoń in Krosno (now State University of Applied Sciences in Krosno).

It has been proposed that the oils characterised in Fig 1 be used to modify lining leather to impart to it antimicrobial properties, i.e., activity against microorganisms inhabiting footwear, potentially pathogenic to humans, which can cause infections and diseases of the foot skin as well as contributing to the biodeterioration of the footwear material [39,40]. The method developed for finishing leather using essential oils with antimicrobial properties has been patented under the title ‘*Method of imparting antimicrobial activity to leather*’ [41].

### Determination of heavy metals in essential oils using atomic absorption spectrometry

The quantitative determination of the contents of the five heavy metals, i.e., arsenic (As), cadmium (Cd), copper (Cu), nickel (Ni) and lead (Pb) in cinnamon, eucalyptus, oregano, manuka and thyme essential oils, characterised in subsection ‘*Essential oils tested for the presence of heavy metals’*, was carried out using an analytical technique, i.e., atomic absorption spectrometry (AAS). These analyses were carried out at the Laboratory of the Institute of Quality Sciences and Product Management of the Krakow University of Economics. In order to determine the contents of the above-listed metals in the oil samples, the method of atomisation in a graphite cuvette with Zeeman-effect background correction was used.

**Fig 2:**
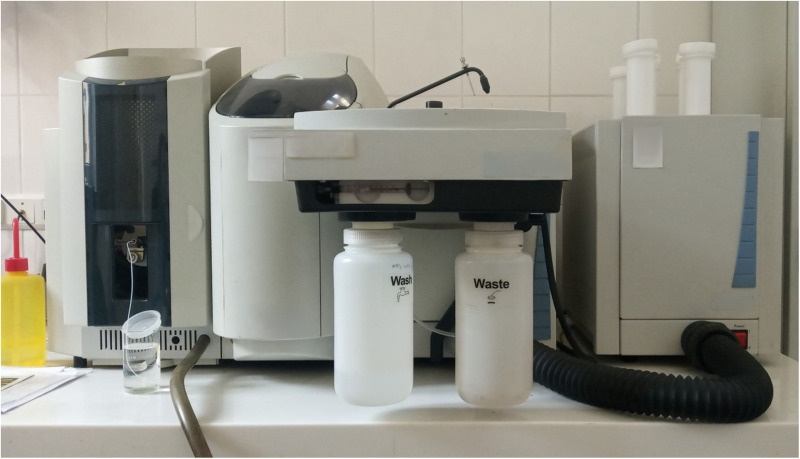
Thermo Scientific^TM^ iCE^TM^ 3000 Series AA Spectrometer. Source: Author’s own research.

Oil samples weighing 0.4 g each were subjected to mineralisation in accordance with a Milestone manual, at 200°C, using a mixture of HNO_3_ (65%) and H_2_O_2_ (30%) (7:1 v/v) in a Milestone Start D microwave unit. The resulting preparations were quantitatively transferred to a volumetric flask and then diluted to 50 mL using demineralised water. The resulting solutions were analysed in a Thermo Scientific^TM^ iCE^TM^ 3000 Series AA Spectrometer (Fig 2) with atomisation in a coated graphite cuvette in a Zeeman furnace. The assays carried out using the method indicated achieved a sensitivity of 0.2 ppb (μg/L) in the solution analysed.

According to the procedures developed by the manufacturer of the Thermo Scientific^TM^ iCE^TM^ 3000 Series AA Spectrometer, it was also necessary to use suitable matrix modifiers, the addition of which allows the optimisation of the analytical conditions. The modifiers were added at a rate of 5 μL per 20 μL of sample volume introduced into the cuvette, which was automatically included in the calculation of the concentrations of the elements in the study. Depending on the type of element to be determined, the following matrix modifiers were used:

- palladium-magnesium Pd + Mg(NO_3_)_2_ for As, Cd and Cu;
- phosphate-magnesium NH_4_H_2_PO_4_ + Mg(NO_3_)_2_ in the case of Pb; and
- magnesium Mg(NO_3_)_2_ in the case of Ni.

The purity of the reagents used during the analyses was verified by preparing a blank sample. This made it possible to determine the possible background and also to include it in the calculation of the contents of the particular heavy metals. In order to quantify the individual elements, a common standard was drawn up, which contained 5.0 μg/L As and Pb, 2.0 μg/L Cu and 1.0 μg/L Cd, respectively.

The analysis of the heavy metal content of the essential oils according to the procedure described in the article was performed in quintuplicate for each type of oil.

### Statistical analysis of the results of heavy metal contents in essential oils

The numerical data, i.e., the results obtained for the contents of Cd, Cu, Ni and Pb in the cinnamon, eucalyptus, oregano, manuka and thyme essential oils, was subjected to statistical analysis using the Statistica 13.3 software. The Fisher-Snedecor F-test was applied in combination with a post-hoc analysis in which comparisons were made using the least significant difference (LSD) test. This made it possible to identify homogeneous groups of arithmetic means of the metal contents of the oils (calculated from 5 measurements for each type of oil – Table in S1 Table). The following research hypotheses were formulated and tested:

- H0: There are no significant differences in heavy metal contents depending on the type of essential oil.
- H1: The contents of heavy metals vary significantly depending on the type of essential oil.

The hypotheses were tested at a significance level of α = 0.05. The decision to accept or reject H0 was made on the basis of the test probability value ‘p-value’, which indicates the lowest significance level at which the H0 would be rejected. It was assumed that p < 0.05 indicates significant variation in the content of a particular heavy metal depending on the type of oil [42]. Statistical analysis was not performed for As because this metal was only detected in two oils, i.e., manuka and cinnamon, and the significant variation in the content of this heavy metal was directly apparent.

## Results and discussion

### Results of studies on the content of heavy metals in essential oils and their statistical analysis

**Fig 3:**
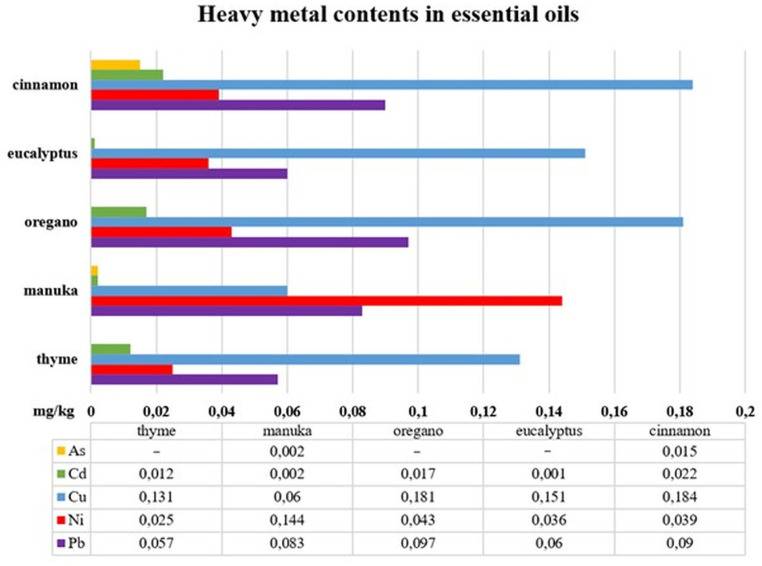
As, Cd, Cu, Ni and Pb contents in cinnamon, eucalyptus, oregano, manuka and thyme essential oils. Source: Author’s own research.

The assays carried out using AAS made it possible to verify whether, and possibly in what quantities, heavy metals, such as As, Cd, Cu, Ni and Pb, are present in the essential oils proposed for the enriching of lining leather, i.e., cinnamon, eucalyptus, oregano, manuka and thyme. The results of the analyses are presented in Fig 3. The figures shown are the arithmetic means of the five measurements for each type of oil (Table in S1 Table).

As can be seen from the data presented in Fig 3, the presence of all the metals in the study was only detected in two of the oils, i.e., cinnamon (from Sri Lanka) and manuka (from New Zealand). In the remaining plant material, i.e., eucalyptus (from Ethiopia), oregano (from Portugal) and thyme (from Turkey) oils, the presence of As was not detected. In the aggregate, it was cinnamon oil that was characterised by the highest heavy metal content, with 0.35 mg/kg As, Cd, Cu, Ni and Pb detected. A slightly lower metal content of 0.338 mg/kg was found for the oregano oil. In manuka and eucalyptus oils, the elements were determined at 0.291 mg/kg and 0.248 mg/kg, respectively. The lowest total content, i.e., 0.225 mg/kg Cd, Cu, Ni and Pb, was found in thyme oil.

When comparing the two oils in which As was detected (Fig 3), cinnamon oil had an almost eight times higher content of this metal than manuka oil (0.015 mg/kg and 0.002 mg/kg respectively). Of the five oils tested, the highest Cd and Cu contents were also found in cinnamon oil (0.022 mg/kg and 0.184 mg/kg respectively). On the other hand, the lowest Cd content (0.001 mg/kg) was found for eucalyptus oil while the lowest content of Cu (0.06 mg/kg) for manuka oil. The analyses showed that Ni was found in the highest amount, i.e., 0.144 mg/kg, in manuka oil while it was found in the lowest amount (0.025 mg/kg) in the thyme oil sample. Lead was determined in the highest amount (0.097 mg/kg) in the oregano oil, and it was found in the lowest amount also in the thyme oil (0.057 mg/kg).

The results obtained using the AAS technique for the heavy metal contents of essential oils (Fig 3) were subjected to a statistical analysis, which was performed using the Fisher-Snedecor F-test combined with a post-hoc analysis based on the LSD test. As can be seen from the data in Table 1, the p-value for the Cd, Cu, Ni and Pb present in varying amounts in the essential oils, is below 0.05. This therefore provides a rationale for rejecting the H0 and accepting the alternative hypothesis, i.e., H1, which states that the contents of heavy metals vary significantly according to the type of essential oil. Post-hoc analysis (LSD test) showed that:

**Table 1:** Results of statistical analysis of the differences in heavy metal contents depending on the type of essential oil.

Heavy metals	Essential oils					
	thyme [n=5]	manuka [n=5]	oregano [n=5]	eucalyptus [n=5]	cinnamon [n=5]	p^b^
	Average content [mg/kg]^a^					
As	not detected	0.002	not detected	not detected	0.015	–
Cd	0.012 b	0.002 a	0.017 c	0.001 a	0.022 d	<0.001
Cu	0.131 b	0.060 a	0.181 d	0.151 c	0.184 d	<0.001
Ni	0.025 a	0.144 c	0.043 b	0.036 b	0.039 b	<0.001
Pb	0.057 a	0.083 b	0.097 c	0.060 a	0.090 b c	<0.001
**Total**	0.225 a	0.291 c	0.338 d	0.248 b	0.350 d	<0.001

^a^different letters next to the mean values of the metal contents of the oils indicate significant differences between them in the LSD test (at α = 0.05);

^b^test probability value.

Source: Author’s own research.

- Cd content is lowest in manuka and eucalyptus oils and highest in cinnamon oil;
- Cu content is lowest in manuka oil and highest in cinnamon and oregano oils;
- Ni content is lowest in thyme oil and highest in manuka oil;
- Pb content is lowest in thyme and eucalyptus oils and highest in oregano oil.

Furthermore, when analyzing the sum of the contents of the heavy metals tested, i.e., As, Cd, Cu, Ni and Pb in the essential oils, it was found to be lowest in thyme oil and highest in oregano and cinnamon oil.

### Analysis of the contents of the selected heavy metals in the essential oils in the context of previous similar experiments

A review of the current literature showed that essential oils are very rarely tested for heavy metals, particularly those types that were investigated in this paper for their potential for enriching lining leather, i.e., cinnamon, eucalyptus, oregano, manuka and thyme, which undoubtedly demonstrates a research gap in this area. This is probably due to the fact that if obtained by properly carried out distillation, they should not contain heavy metals as their molecules do not volatilise under normal distillation conditions due to their high weight and mass [43]. However, metals can enter the oils at further stages of production and/or during storage, e.g., from steel containers [44]. Experiments using the inductively coupled plasma optical emission spectroscopy (ICP-OES) technique to verify the presence of heavy metals in essential oil extracted from the *Eucalyptus globulus* plant species were conducted in 2019 by the Korean researchers Jeong and Lim [45]. The authors did not provide information on the method of obtaining the oil or the country of its production. They showed that As, Cd, Cu, Ni and Pb were present in the oil they studied in amounts <10 mg/kg each. Therefore, it is not possible to compare them with the results of the studies conducted for eucalyptus oil within this publication. In the European essential oil extracted by steam distillation from the plant species *Origanum vulgare ssp. hirtum* (stem, leaves and flowers), proposed for use as a sensory additive in animal feed, the content of selected heavy metals, including As, Cd and Pb, was also determined. According to the authors of the experiment, these metals were below the limit of detection, which is < 0.01 mg/kg, for each of the three batches of the oil tested [46]. In the Portuguese oregano oil analysed in this paper As was also not detected, but the oil contained 0.017 mg/kg Cd and 0.097 mg/kg Pb (Fig 3). In 2024 Croatian researchers produced an essential oil by hydrodistillation from the plant *Origanum vulgare* L. and tested it for heavy metal content. The plant material was purchased from a local store and came from the EU according to the information provided by the authors. Analyses carried out using an inductively coupled plasma quadrupole mass spectrometer (ICP-Q-MS) allowed the determination of the following metal contents in the oil: As – 0.38 mg/kg; Cd – 0.02 mg/kg; Cu – 21.44 mg/kg; Ni – 1.19 mg/kg and Pb – 0.82 mg/kg [47]. When comparing the indicated data with the information in Fig 3, it can be seen that the oregano oil, which was tested in the context of its applicability for the modification of leather for the inner parts of footwear, not only does not contain As, but also has a lower content of the other metals analysed. A particularly large difference was observed for Cu and Ni. In 2022, Iordache et al. [44] determined heavy metals in several obtained from the plant *Thymus vulgaris* oils produced in different countries, by different manufacturers, available on the Romanian market, while at the same time assessing the health risk. The study was conducted using the inductively coupled plasma mass spectrometry (ICP-MS) technique. The highest heavy metal contents detected in the oils were: As – 0.011 mg/kg – in oil from UE; Cd – 0.014 mg/kg – in oil from Romania; Cu – 0.37 mg/kg – in oil from India; Ni – 1.44 mg/kg – in oil from Salt Lake City, UT, USA, and Pb – 0.050 mg/kg – in oil from Romania. When comparing the values indicated with the results obtained for Turkish thyme oil proposed for enriching lining leather (Fig 3), it can be seen that it contained lower amounts of Cd, Cu, Ni and Pb; additionally, the presence of As was not detected in the oil. Deliorman Orhan, Acar and Alp [48], used the ICP-MS technique and carried out analyses of the content of, inter alia, Cd and Pb in a thyme essential oil, which was also acquired in Turkey and sold as useful for aromatherapy. This oil contained 7.41 ± 0.93 ng/g (0.00741 ± 0.00093 mg/kg) of Cd while Pb could not be determined (below the limit of detection). The thyme oil tested within the scope of the present paper contained these metals in higher amounts, i.e., Cd – 0.012 mg/kg and Pb – 0.057 mg/kg.

There can be found reports in the world literature on other essential oils in which heavy metal levels were determined. For instance, Zheljazkov and Jekov [49] investigated the contents of, inter alia, As, Cd, Cu and Pb in commercially available Bulgarian essential oils from *Rosa damascena var trigintipetala*, *Lavandula vera DC*, *Mentha piperita* L., *Mentha arvensis* L., *Salvia sclarea* L., *Ocimum basilicum* L., *Foeniculum officinale All*, *Coriandrum sativum* L., *Anethum graveolens* L., *Hyssopus officinalis* L. and *Rhus cotinus* L. For this purpose, the scientists used graphite furnace atomic absorption (GFAA) and inductively coupled plasma (ICP) techniques. For all the oils mentioned, As levels were <0.08 mg/L. On comparison, in the manuka oil from New Zealand it was 0.002 mg/kg, in the cinnamon oil from Sri Lanka it was determined to be 0.015 mg/kg (Fig 3), while it was not detected in the other oils. Zheljazkov and Jekov [49] did not identify the presence of Cd in any of the oils tested. In the study concerned (Fig 3), it was present in amounts ranging from 0.001 mg/kg (eucalyptus oil from Ethiopia) to 0.022 mg/kg (cinnamon oil from Sri Lanka). The Cu content of the Bulgarian oils ranged from 0.013 mg/L (*Rosa damascena var trigintipetala* oil) to 0.092 mg/L (*Mentha piperita* L. oil). Of the oils tested by the author, only manuka oil from New Zealand had a lower content of this heavy metal (0.06 mg/kg); in the other oils it was present in amounts above 0.1 mg/kg (Fig 3). Zheljazkov and Jekov [49] determined the lowest Pb content, i.e., < 0.001 mg/L, for oil from the plant *Rosa damascena var trigintipetala* while the highest, i.e., 0.021 mg/L, for oil from *Anethum graveolens* L. As can be seen from the data in Fig 3, all of the oils tested for enriching lining leather had a higher content of this heavy metal (ranging from 0.057 mg/kg for thyme oil from Turkey to 0.097 mg/kg for oregano oil from Portugal). In 2020 Angelova [50], who also used ICP, determined the content of, inter alia, Cd and Pb in essential oils extracted by steam distillation from the plant species *Melissa officinalis* L. growing on plots located at different distances from the Non-Ferrous-Metal Works (Bulgaria). In both oils, irrespective of the distance from the smelter to where the plants were grown, no Cd content was detected (below the limits of the quantitative measurement). However, the Pb content was at 0.2 mg/kg (oil S1 from a plant growing 0.5 km from the plant) and 0.03 mg/kg (oil S2 from a plant growing 15 km from the plant), which confirmed the susceptibility of the plant to the accumulation of this heavy metal. The essential oils tested within the scope of the present paper contained Pb ranging from 0.057 mg/kg (thyme oil from Turkey) to 0.097 mg/kg (oregano oil from Portugal) (Fig 3). Thus, they were characterised by lower levels of this metal compared with the S1 oil studied by Angelova [50] and, at the same time, higher levels compared with the S2 oil.

Heavy metals were much more frequently determined by researchers in samples of various plant species, including those from which essential oils are produced. In cinnamon, which is a spice extracted from the plant species *Cinnamomum verum* (synonymous with *Cinnamomum zeylanicum*), the presence of some selected heavy metals was verified by [51–57]. Heavy metals were detected in the leaves of *Eucalyptus globulus*, e.g., by Youssef [58] and Zárate-Quiñones et al. [59]. They were detected by Meister et al. [60] in the plant foliage of *Leptospermum scoparium*, in manuka honey made from this raw material, and by Prosser [61] in shoot and leaf samples of this plant. These elements were determined in samples obtained from the plant species *Origanum vulgare* by Behmen et al. [62], Dghaim et al. [63], Dogan et al. [64] or Marinescu et al. [65]. The plant *Thymus vulgaris* was also frequently used as test material in which the presence of heavy metals was verified. Such experiments were conducted by, e.g., Djarmouni et al. [6], Dghaim et al. [63], Marinescu et al. [65], Abu-Darwish [66], Abu-Darwish and Abu-Dieyeh [67], Akoury et al. [68], Al-Keriawy et al. [69] or Karagözoğlu and Kiran [70].

Variation in the chemical composition of essential oils is common. As pointed out by Fokou et al. [71] oils extracted from different plants belonging to the same species may have different compositions. The chemical profile of the oil is influenced by many factors, both biotic and abiotic, as well as the post-harvest treatment of the plants, methods of oil extraction or conservation conditions. By way of example, contaminated soil as well as polluted irrigation is cited as the main cause of heavy metal contamination with regard to agricultural thyme from which thyme oil is extracted. A similar relation was shown Angelova [50], cited earlier, who confirmed that essential oil extracted from *Melissa officinalis* L. growing closer to the metal smelter contained more Pb compared with oil produced from plants growing further away from the plant.

### Assessment of the possible impact of the application of essential oils to lining leather on footwear quality and consumer safety

Heavy metals, including those determined in essential oils within the framework of this research, are also usually present in varying amounts in leather that is used in the manufacture of a wide range of goods, including footwear. This is a result of, among other things, the application of chemicals normally used in the tanning industry to obtain a raw material with the desired properties. The occurrence of heavy metals in leather is confirmed by analyses carried out by, e.g., Bielak and Marcinkowska [23], Beik et al. [72], Germer et al. [73] or Scheffler and Pozebon [74]. The contents of As, Cd, Cu, Ni and Pb detected by the above-mentioned researchers in various types of leather, including those for footwear, are shown in Table 2. Depending on the type of element, they can be present in very different and often significant amounts. Thus, natural essential oils introduced into the leather at the stage of fatliquoring should be characterised by the absence or sufficiently low contents of heavy metals so that they do not cause their accumulation in the material, which could have a negative impact on its quality and the safety of the consumer using the product made from such raw material.

**Table 2:** Investigation of As, Cd, Cu, Ni and Pb content in leathers.

Leather type/characteristics	Heavy metals	Content [mg/kg]	Analysis technique	Reference
**Leathers that can be used for footwear components**				
**floater leather**	Cd	0.20 ± 0.10	USN-ICP OES^c^	Scheffler and Pozebon [74]
	Cu	130 ± 14		
	Ni	1.20 ± 0.10		
	Pb	<0.10^b^		
**shoe leather**	Cd	0.20 ± 0.04		
	Cu	12.0 ± 1.5		
	Ni	0.65 ± 0.05		
	Pb	0.30 ± 0.15		
**nappa leather**	Cd	0.21 ± 0.04		
	Cu	5.30 ± 0.60		
	Ni	0.81 ± 0.15		
	Pb	4.0 ± 0.90		
**goat, aniline, soft, chrome leather for shoe lining**	As	0.005	AAS	Bielak and Marcinkowska [23]
	Cd	0.026		
	Cu	4.4		
	Pb	0.004		
**pig, grain, soft, chrome leather for shoe lining**	As	not detected		
	Cd	0.009		
	Cu	6.1		
	Pb	0.005		
**cowhide, nubuck, chrome leather for shoe uppers**	As	not detected		
	Cd	0.009		
	Cu	3.2		
	Pb	0.003		
**Leathers for which no information on the intended use is given**				
**six leathers of different kinds of manufacture** ^a^	Pb	depending on the sample preparation procedure: <4^b^ – 2240 ± 200 <9^b^ – 2490 ± 80 <6^b^ – 2370 ± 120	AAS	Beik et al. [72]
**eighteen leathers of different kinds of manufacture** ^a^	Cd	<0.3^b^ – 53.19 ± 1.28	ICP-MS	Germer et al. [73]
	Cu	<0.2^b^ – 484.61 ± 4.33		
	Pb	<0.02^b^ – 211.31 ± 0.50		

^a^the article lacks a detailed characterization of the tested leathers;

^b^below the limit of detection; ^c^ ultrasonic nebulization axial view inductively coupled plasma optical emission spectrometry.

Source: Author’s own research.

An indicator of the good quality and safety of a product will be its compliance with specific requirements with regard to the contents of harmful substances. Limits for selected hazardous chemicals, including heavy metals, that may appear in leather products have been developed by the International Association for Research and Testing in the Field of Textile and Leather Ecology (Oeko-Tex®) and are included in the Oeko-Tex® Leather Standard [36]. The Oeko-Tex® Leather Standard label certifies that the product is harmless to human health while the certification itself is entirely voluntary, like many others, such as the EU Ecolabel [75]. Another document setting out the limits with regard to, inter alia, the presence of heavy metals in products is Regulation (EC) No 1907/2006 of the European Parliament and of the Council of 18 December 2006 concerning the Registration, Evaluation, Authorisation and Restriction of Chemicals (REACH), establishing a European Chemicals Agency, amending Directive 1999/45/EC and repealing Council Regulation (EEC) No 793/93 and Commission Regulation (EC) No 1488/94 as well as Council Directive 76/769/EEC and Commission Directives 91/155/EEC, 93/67/EEC, 93/105/EC and 2000/21/EC [35]. Table 3 shows the limits set out in Oeko-Tex® Leather Standard [36] and REACH Regulation [35] with regard to As, Cd, Cu, Ni and Pb concerning their quantity determined after extraction as well as their total contents in the product. When comparing the results obtained for the contents of these metals in cinnamon, eucalyptus, oregano, manuka and thyme essential oils (Fig 3) with the limits specified in the Oeko-Tex® Leather Standard [36] and REACH Regulation [35], it can be seen that the plant material tested contains heavy metals in amounts well below the limits allowed by these documents for various non-food products. In terms of the Oeko-Tex® Leather Standard [36], this applies to the limits specified for all product classes, i.e., I – products for babies, II – products with direct contact to skin, III – products without direct contact to skin, and IV – decoration material. However, in the context of the proposed application of the above-mentioned oils, i.e., to be used for the enriching of leather for the inside parts of footwear, the assessment should take into account first and foremost the limits set for Classes I and II.

**Table 3:** Limits for As, Cd, Cu, Ni and Pb content in products/materials according to Oeko-Tex® Leather Standard and REACH Regulation.

Limit values according to Oeko-Tex® Leather Standard [36]				
Heavy metals	I Products for babies	II Products with direct contact to skin	III Products without direct contact to skin	IV Decoration material
	less than, [mg/kg]			
**extractable**				
As	0.2	1.0		
Cd	0.1			
Cu	25.0	50.0		
Ni	1.0	4.0		
Pb	0.2	1.0		
**total content**				
As	100			
Cd	40.0			
Cu	–			
Ni	–			
Pb	90.0			
**Limit values according to REACH Regulation [35]**				
**Heavy metals**	**Concentration limit by weight after extraction, [mg/kg]**			
Arsenic compounds	1 (expressed as As metal that can be extracted from the material)			
Cd and its compounds	1 (expressed as Cd metal that can be extracted from the material)			
Cu	–			
Ni	–			
Pb and its compounds	1 (expressed as Pb metal that can be extracted from the material)			

Source: Author’s own research based on Oeko-Tex® Leather Standard [36] and REACH Regulation [35].

Guidelines on the recommended content of harmful chemicals, including heavy metals that may occur in apparel and footwear have also been created by the Apparel and Footwear International RSL Management Working Group (AFIRM Group), founded in 2004. Its activities are focused on limiting the use and the impact of these substances in the supply chain of these product groups. The limits are contained in the so-called *Restricted Substances List* developed by the AFIRM Group to support supply chain participants in their efforts to improve product quality and safety or reduce the environmental impact of apparel and footwear [37]. The maximum heavy metal contents of products as recommended by the AFIRM Group, which metals were also determined in essential oils within the scope of this publication, are shown in Table 4. For the most part, they are in line with the limits set out in the Oeko-Tex® Leather Standard [36], i.e., identical limits for Cd, Cu and Pb (extractable) as well as As, Cd and Pb (total content) can be found in both documents. In the case of As and Ni (extractable), the *Restricted Substances List* document [37] contains stricter limits than the Oeko-Tex® Leather Standard [36], as after extraction the contents of these elements should be less than 0.2 mg/kg and 1 mg/kg, respectively, for products intended for babies and children as well as those for adults. In the Oeko-Tex® Leather Standard [36] such limits only apply to products for babies while for the rest, i.e., those in direct as well as without direct contact with the skin and decoration material, higher contents of these metals are permissible (As <1 mg/kg, Ni < 4 mg/kg). When comparing the contents of As, Cd, Cu, Ni and Pb in the essential oils under analysis (Fig 3) with the permissible limits for these metals indicated in the *Restricted Substances List* ([37], Table 4), it can be concluded that the plant material tested is characterised by the presence of these heavy metals at a very low level.

**Table 4:** Limits for As, Cd, Cu, Ni and Pb content in products/materials according to *Restricted Substances List* and *List of Restricted Substances in Shoes.*

Limit values according to *Restricted Substances List* [37]		
Heavy metals	Products for babies and children	Products for adults
	less than, [ppm = mg/kg]	
**extractable**		
As	0.2	
Cd	0.1	
Cu	25	50
Ni	1	
Pb	0.2	1
**total content**		
As	100	
Cd	40	
Cu	–	
Ni	–	
Pb	90	
**Limit values according to *List of Restricted Substances in Shoes* [38]**		
**Heavy metals**	**Materials with skin contact (only)**	**Materials with and without skin contact**
	**[mg/kg]**	
**soluble**		
As	0.2^a^^-^^c^	–
Cd	0.1^a^^-^^c^	–
Cu	25^a^^-^^d^	–
Ni	4.0^a^^-^^c^	–
Pb	1.0^a^^-^^c^	–
**total**		
As	–	–
Cd	–	100^b^/ 40^b^^,^^e^
Cu	–	–
Ni	–	–
Pb	–	90^b^

^a^applies to leather/ fur;

^b^applies to coated leather;

^c^applies to leather fibre board,

^d^for Arab countries,

^e^for USA only.

Source: Author’s own research based on *Restricted Substances List* [37] and *List of Restricted Substances in Shoes* [38].

It is worth noting that the AFIRM Group has in recent years also developed the so-called Risk Matrix where information and guidance can be obtained on the risks associated with the presence of hazardous chemicals in various materials. This study supports brands and suppliers to effectively manage chemical risks by adopting a common approach to testing. When analysing the Risk Matrix in terms of the juxtaposition of ‘*Heavy metals*’ as ‘*substance*’ and ‘*Natural Leather & Fur Skin*’ as ‘*material*’ in relation to heavy metals (extractable), the red code is used, which means ‘*Higher risk*’ and ‘*Testing required*’, but for heavy metals (total) the code is orange, meaning ‘*Lower risk*’ and ‘*Testing recommended and may be required at brand discretion*’ [37]. These entries are therefore indicative of the fact that the presence of heavy metals in natural leather products is an important issue and should be subject to verification.

The problems of the quality of footwear and leather products are dealt with by the German association Cooperation for Assuring Defined Standards for Shoe and Leather Goods Production e.V. (CADS) whose activities include, among other things, promoting the production and marketing of sustainable, environmentally friendly and toxic-free footwear materials and footwear [76]. In view of the fact that different countries may have different guidelines for the permissible contents of hazardous chemicals in the products concerned, the association set minimum requirements and developed the *List of Restricted Substances in Shoes* [38] which also included heavy metals. The limits for heavy metals in force in the European Union, included in this list, which were also found in cinnamon, eucalyptus, oregano, manuka and thyme essential oils, are listed in Table 4. For As and Cu (soluble), the limits proposed by the CADS [38] for leather/fur, coated leather and leather fibre board that are in contact with the skin are 0.2 mg/kg and 25 mg/kg, respectively. Oeko-Tex® Leather Standard [36] recommends that exactly the same values are not exceeded in products for babies while the requirements for those that are in direct contact with the skin are less stringent (As <1.0 mg/kg, Cu < 50.0 mg/kg). It should be noted, however, that the limit set by the CADS Association for Cu applies for Arab countries. According to the *List of Restricted Substances in Shoes* [38], the content of Cd (soluble) in leather materials with skin contact should be a maximum of 0.1 mg/kg while the limit <0.1 mg/kg according to Oeko-Tex® Leather Standard [36] applies to products for babies, products with and without direct contact to skin. The maximum contents of Ni and Pb (soluble) in leather/fur, coated leather and leather fibre board with skin contact is 4.0 mg/kg and 1.0 mg/kg, respectively, according to the *List of Restricted Substances in Shoes* [38]. Oeko-Tex® Leather Standard [36] assumes that the values indicated for these elements should not be exceeded not only in products with direct skin contact, but also in those without direct contact. The *List of Restricted Substances in Shoes* [38] also includes guidelines for the permissible total contents of Cd and Pb in coated leather with and without skin contact. The limit recommended by the CADS Association for Cd, is 100 mg/kg (except in the USA where a value of 40 mg/kg applies) and this is more than twice as high as that proposed for leather products by Oeko-Tex® Leather Standard [36], i.e., < 40.0 mg/kg. In contrast, the limits indicated by both documents with regard to Pb are very similar. The CADS Association proposes a maximum content of 90 mg/kg [38] for this metal in coated leather while the Oeko-Tex® Association allows a total Pb content in leather products lower than 90 mg/kg [36]. When comparing the results obtained for the contents of As, Cd, Cu, Ni and Pb in the essential oils proposed for the modification of lining leather (Fig 3) with the limits for these metals listed in the *List of Restricted Substances in Shoes* ([38], Table 4), it can be noted that all the oils tested are characterised by very low contents of these metals.

**Fig 4:**
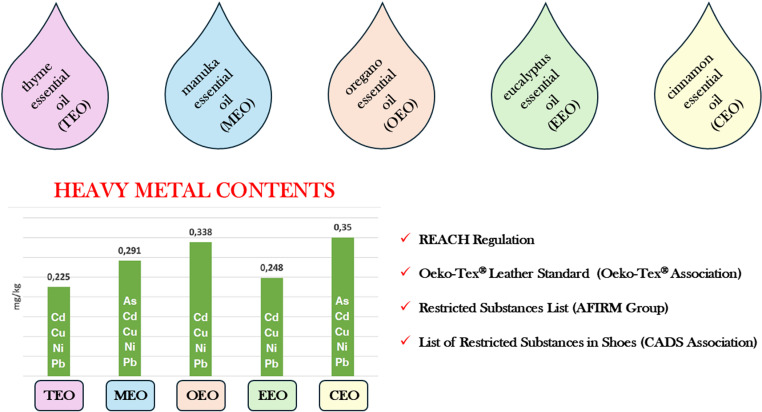
Heavy metal contents in essential oils in light of requirements – summary. Source: Author’s own research.

The analysis of the European REACH Regulation [35], Oeko-Tex® Leather Standard by Oeko-Tex® Association [36], *Restricted Substances List* by AFIRM Group [37] and *List of Restricted Substances in Shoes* by CADS Association [38] in terms of the recommended limits for heavy metals in products made it possible to assess the possible impact of the addition of essential oils proposed for the enriching of leather for the inside parts of footwear on the quality and safety of this raw material. As the contents of As, Cd, Cu, Ni and Pb in cinnamon, eucalyptus, oregano, manuka and thyme oils are very low (Fig 4) compared with the limits, it can be concluded that their introduction into lining leather will not cause a risk related to the accumulation of heavy metals in the material. Therefore, the application of natural substances with antimicrobial properties to leather, which is the test material, should not impair the quality of footwear made from such a modified raw material as understood in the context of consumer safety.

## Summary and conclusions

Due to the fact that footwear is an essential part of people’s clothing, which they use every day in a wide variety of conditions and activities, it must not pose any health risks. This applies to the product as a whole as well as to its individual parts, especially those in close or direct contact with the wearer’s body, an example of which are the components lining the inside of the shoe. They should not contain toxic substances in quantities exceeding the permissible limits as they may adversely affect the wearer of the footwear.

The tests conducted using atomic absorption spectrometry for the contents of the heavy metals: arsenic, cadmium, copper, nickel and lead in the essential oils proposed for the modification of lining leather, showed that:

- the metal present in the lowest amount in the plant materials tested was arsenic, which was only detected in cinnamon and manuka oils;
- the metal present in the lowest amount in cinnamon oil was arsenic (0.015 mg/kg); in the eucalyptus, oregano and thyme oils it was cadmium (0.001 mg/kg, 0.017 mg/kg, 0.012 mg/kg respectively); also in the manuka oil the lowest amount identified was that of cadmium as well as of arsenic (0.002 mg/kg each);
- the metal present in highest quantity in cinnamon, eucalyptus, oregano and thyme oils was copper (0.184 mg/kg, 0.151 mg/kg, 0.181 mg/kg, 0.131 mg/kg respectively) while nickel was detected in highest quantity in the manuka oil (0.144 mg/kg).

In addition, the statistical analysis of the results, conducted using the Fisher-Snedecor F-test in combination with a post-hoc analysis based on the LSD test, confirmed that the heavy metal content varied significantly depending on the type of essential oil. Using the LSD test, it was shown that:

- cadmium content is lowest in manuka and eucalyptus oils, and highest in cinnamon oil;
- copper content is lowest in manuka oil and highest in cinnamon and oregano oils;
- nickel content is lowest in thyme oil and highest in manuka oil;
- lead content is lowest in thyme and eucalyptus oils and highest in oregano oil.

Furthermore, when the sum of the contents of the heavy metals tested, i.e., As, Cd, Cu, Ni and Pb in the essential oils was analysed, it was found to be lowest in thyme oil and highest in oregano and cinnamon oils.

When comparing the results of the analysis of heavy metal contents in the plant-based test material with the permissible limits for their contents in products, as specified in the European REACH Regulation [35], Oeko-Tex® Leather Standard by Oeko-Tex® Association [36], *Restricted Substances List* by AFIRM Group [37] and *List of Restricted Substances in Shoes* by CADS Association [38], it was found that cinnamon, eucalyptus, oregano, manuka and thyme essential oils were characterised by the presence of arsenic, cadmium, copper, nickel and lead at very low levels. Therefore, the modification of cowhide leather intended for the inside parts of footwear by applying the above-mentioned oils during the fatliquoring stage to improve the hygienic properties of the product should not have a detrimental effect on its quality as understood in the context of safety for the user. However, this will not be the case if other substances are used during the leather manufacturing process, the addition of which may cause a significant increase in the heavy metal contents of the material. A literature review confirmed that some of these elements may be present in leather raw material in significant quantities.

A research gap was encountered when tackling the subject of verifying the presence of arsenic, cadmium, copper, nickel and lead in essential oils selected for the purpose of enriching lining leather. In view of the very limited number of analyses of these heavy metal contents of cinnamon, eucalyptus, oregano, manuka and thyme essential oils, as well as their various applications in both food and non-food products, in the context of the studies carried out and the results obtained, which, although small in number, nevertheless confirmed the presence of these metals in the oils, it is reasonable to conclude that such studies should be carried out on a wider scale. Due to various factors and, above all, their natural origin, essential oils may have different parameters and compositions. Therefore, in order to ensure the quality and safety of products enriched with them, especially those the use of which will expose the consumer to prolonged and direct contact, it is necessary to continuously monitor their important property, which is the possible content of harmful heavy metals.

It is worth mentioning that there are reports in the literature of the use of essential oils for various applications in leather processing, mainly as natural biocides. However, there is a lack of information regarding their heavy metal contents or research in this area. Undoubtedly, this also confirms the identification of a research gap and the novelty of the research presented in which the quality and safety of essential oils were assessed in the context of their possible unconventional use to improve the hygienic properties of footwear.

There are plans for multifaceted and in-depth research into the potential use of natural essential oils for the enrichment of leather in the near future. Consideration will be given to further types of oils obtained from other regions of the world. The possible impact of various factors (e.g., temperature and chemical tanning agents) during the leather processing or footwear production on the quality and safety of essential oils will also be examined. Finally, the leathers themselves will be tested for harmful heavy metals after having been treated with essential oils.

## Supporting information

S1 Table As, Cd, Cu, Ni and Pb content in the cinnamon, eucalyptus, oregano, manuka and thyme essential oils. Source: Author’s own research.
(DOCX)
