# Supplementary material for: Heavy metals in essential oils proposed for the modification of footwear lining leather—Quality management and product safety
Source: PLoS One. 2025 Jun 26;20(6):e0325766. doi: 10.1371/journal.pone.0325766 (PMC12200777; doi:10.1371/journal.pone.0325766)
Supplement: S1 Table — Source: Author’s own research. (DOCX) [file pone.0325766.s001.docx]

**S1 Table. As, Cd, Cu, Ni and Pb content in the cinnamon, eucalyptus, oregano, manuka and thyme essential oils.**

| **Heavy metals** | **Sample no.** | **Content detected in essential oils** | | | | |
| --- | --- | --- | --- | --- | --- | --- |
|  |  | **thyme** | **manuka** | **oregano** | **eucalyptus** | **cinnamon** |
|  |  | **[mg/kg]** | | | | |
| **As** | **1** | not detected | 0.001 | not detected | not detected | 0.018 |
|  | **2** | not detected | 0.001 | not detected | not detected | 0.016 |
|  | **3** | not detected | 0.002 | not detected | not detected | 0.015 |
|  | **4** | not detected | 0.004 | not detected | not detected | 0.012 |
|  | **5** | not detected | 0.002 | not detected | not detected | 0.014 |
| **Arithmetic mean** | | **-** | **0.002** | **-** | **-** | **0.015** |
| **Cd** | **1** | 0.011 | 0.002 | 0.021 | 0.001 | 0.022 |
|  | **2** | 0.015 | 0.001 | 0.018 | 0.0015 | 0.025 |
|  | **3** | 0.012 | 0.002 | 0.015 | 0.001 | 0.021 |
|  | **4** | 0.012 | 0.003 | 0.014 | 0.001 | 0.021 |
|  | **5** | 0.010 | 0.002 | 0.017 | 0.0005 | 0.021 |
| **Arithmetic mean** | | **0.012** | **0.002** | **0.017** | **0.001** | **0.022** |
| **Cu** | **1** | 0.136 | 0.061 | 0.181 | 0.151 | 0.184 |
|  | **2** | 0.133 | 0.062 | 0.179 | 0.150 | 0.183 |
|  | **3** | 0.129 | 0.058 | 0.184 | 0.153 | 0.184 |
|  | **4** | 0.126 | 0.060 | 0.183 | 0.151 | 0.186 |
|  | **5** | 0.131 | 0.059 | 0.178 | 0.149 | 0.183 |
| **Arithmetic mean** | | **0.131** | **0.060** | **0.181** | **0.151** | **0.184** |
| **Ni** | **1** | 0.025 | 0.144 | 0.045 | 0.036 | 0.039 |
|  | **2** | 0.023 | 0.141 | 0.043 | 0.036 | 0.039 |
|  | **3** | 0.024 | 0.144 | 0.047 | 0.034 | 0.041 |
|  | **4** | 0.028 | 0.149 | 0.040 | 0.036 | 0.038 |
|  | **5** | 0.025 | 0.142 | 0.040 | 0.038 | 0.038 |
| **Arithmetic mean** | | **0.025** | **0.144** | **0.043** | **0.036** | **0.039** |
| **Pb** | **1** | 0.054 | 0.083 | 0.093 | 0.060 | 0.090 |
|  | **2** | 0.057 | 0.081 | 0.101 | 0.060 | 0.095 |
|  | **3** | 0.061 | 0.083 | 0.095 | 0.060 | 0.088 |
|  | **4** | 0.056 | 0.085 | 0.097 | 0.062 | 0.087 |
|  | **5** | 0.057 | 0.083 | 0.099 | 0.058 | 0.090 |
| **Arithmetic mean** | | **0.057** | **0.083** | **0.097** | **0.060** | **0.090** |

Source: Author’s own research.
